# Quantum magnetic imaging of iron organelles within the pigeon cochlea

**DOI:** 10.1073/pnas.2112749118

**Published:** 2021-11-15

**Authors:** Robert W. de Gille, Julia M. McCoey, Liam T. Hall, Jean-Philippe Tetienne, E. Pascal Malkemper, David A. Keays, Lloyd C. L. Hollenberg, David A. Simpson

**Affiliations:** ^a^School of Physics, University of Melbourne, Parkville, 3010 VIC, Australia;; ^b^School of Chemistry, University of Melbourne, Parkville, 3010 VIC, Australia;; ^c^Max Planck Research Group Neurobiology of Magnetoreception, Center of Advanced European Studies and Research, 53175 Bonn, Germany;; ^d^Institute of Molecular Pathology (IMP), Vienna Biocenter (VBC), 1030 Vienna, Austria;; ^e^Department of Anatomy and Neuroscience, University of Melbourne, Parkville, 3010 VIC, Australia;; ^f^Division of Neurobiology, Department Biology II, Ludwig-Maximilians-University Munich, Planegg-Martinsried 82152, Germany

**Keywords:** diamond, magnetoreception, avian, cochlea, quantum sensing

## Abstract

The ability of pigeons to sense geomagnetic fields has been conclusively established despite a notable lack of determination of the underlying biophysical mechanisms. Quasi-spherical iron organelles previously termed “cuticulosomes” in the cochlea of pigeons have potential relevance to magnetoreception due to their location and iron composition; however, data regarding the magnetic susceptibility of these structures are currently limited. Here quantum magnetic imaging techniques are applied to characterize the magnetic properties of individual iron cuticulosomes in situ. The stray magnetic fields emanating from cuticulosomes are mapped and compared to a detailed analytical model to provide an estimate of the magnetic susceptibility of the individual particles. The images reveal the presence of superparamagnetic and ferrimagnetic domains within individual cuticulosomes and magnetic susceptibilities within the range 0.029 to 0.22. These results provide insights into the elusive physiological roles of cuticulosomes. The susceptibilities measured are not consistent with a torque-based model of magnetoreception, placing iron storage and stereocilia stabilization as the two leading putative cuticulosome functions. This work establishes quantum magnetic imaging as an important tool to complement the existing array of techniques used to screen for potential magnetic particle–based magnetoreceptor candidates.

## Significance

Cuticulosomes are subcellular structures located within the inner ear hair cells of a variety of avian species with potential relevance to magnetoreception. Here we apply quantum magnetic microscopy to image the magnetic properties of individual iron cuticulosomes within tissue samples. The magnetic susceptibility of the cuticulosomes was determined by characterizing the stray magnetic field strength as a function of applied magnetic field in two distinct locations of the pigeon inner ear. The measured susceptibilities do not support the particle model of magnetoreception, suggesting the physiological relevance of cuticulosomes lies in iron storage or stabilization of stereocilia. The quantum magnetic imaging method can be applied across a variety of biological systems providing an effective tool to screen for magnetic particle–based magnetoreceptors.

Magnetoreception in diverse avian species including pigeons has been well established through behavioral studies (1); however, the biophysical mechanisms that underlie the sense are yet to be determined (2). The models proposed to date are based on three biophysical concepts: 1) a torque-based ferrimagnetic particle model (3), 2) the formation of radical pairs (4), and 3) electromagnetic induction (5). Magnetoreceptor candidates based on these models exist in the retina (4) and the inner ear (6). Analysis of the immediate early gene C-FOS has implicated the vestibular system in the magnetic sense, which in turn led to the discovery of cuticulosomes (7). These spherical structures reside in the actin-rich cuticular plate of hair cells in the lagena, basilar papilla, saccule, and utricle of the pigeon inner ear (6). Cuticulosomes have been studied extensively using high-resolution electron microscopy to characterize their size and morphology (6). The available evidence indicates that they are primarily composed of the iron oxide ferrihydrite, are ∼300 to 600 nm in diameter, and are present in 2 to 6% of vestibular hair cells and ∼25% of acoustic hair cells located in the basilar papilla. The function of these organelles is unknown, but it has been proposed that they either act as a store for excess iron, stabilize stereocilia, or mediate magnetoreception.

The acquisition of empirical magnetic data from cuticulosomes poses a significant technological challenge due to the small size and magnetization of these objects (8). The spatial resolution of high-temperature scanning superconducting quantum interference devices is insufficient to image single magnetic structures of this size, despite their high magnetic sensitivity (9). Spatial resolutions on nanometer scales can be achieved using magnetic force microscopy and scanning tunneling microscopes. These techniques are of limited utility to the current application due to long acquisition times, limited measurement throughput, and in some cases cryogenic and high-vacuum environments (10, 11). Elemental analysis has been undertaken using X-ray fluorescence microscopy (12) and inductively coupled plasma mass spectrometry (13). However, direct measurements of the magnetic properties of these particles are not provided through the application of these technologies.

## Results and Discussion

**Fig. 1:**
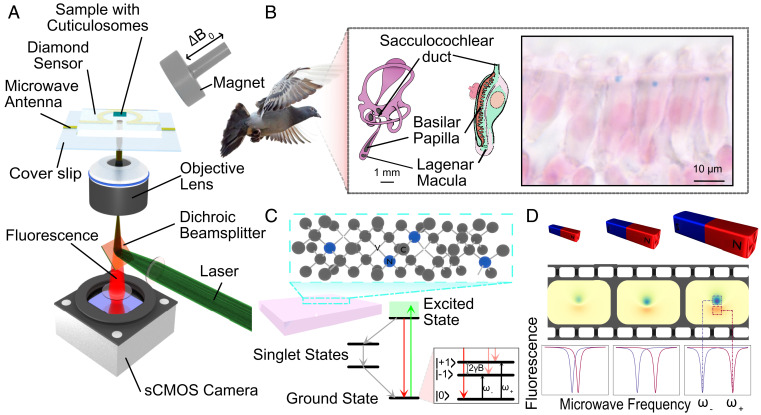
Overview of the design of the experiment. (*A*) The biological sample is affixed to the top of a diamond imaging chip containing a layer of NVs. The imaging chip sits on top of a glass coverslip onto which a gold microwave antenna is printed. The NV centers are addressed using a 532-nm excitation wave, and their fluorescence is captured using an sCMOS camera. A permanent magnet on a mobile mount is used to control the applied magnetic field at the NV layer. (*B*) Details of the biological sample of interest. Thin sections are taken containing inner ear hair cells from the lagena and basilar papilla of pigeons. The diagrams of the pigeon inner ear and cochlea show the region of the pigeon anatomy of interest in these experiments. The section displayed contains structures which are stained using Prussian blue and is representative of the sections and structures of interest in this study. The sections analyzed were not subject to Prussian blue staining. (*C*) Details of the diamond imaging chips used. The NV centers fluoresce red when optically addressed with the 532-nm excitation wave. A nonfluorescent decay pathway linked to the ∣+1〉 spin sublevels of the excited state manifold results in a reduced NV fluorescence. Additionally, the nonfluorescent decay pathway populates the ∣0〉 sublevel of the ground state manifold. NV systems in the ∣0〉 sublevel can be driven into the other sublevels through the application of a resonant microwave field, *ω*. The combination of these properties allows spin state control and readout. The positions of the ∣−1〉 and ∣+1〉 spin sublevels split in frequency space as a magnetic field is applied along the axis of the NV system. (*D*) Film reel showing magnetic field maps of a magnetic particle with increasing applied magnetic field strengths. The images are produced by measuring the fluorescence of the NV centers at each pixel and sweeping the microwave frequency. Where the frequency is resonant with the ∣0〉 to ∣−1〉 transition, a dip in the fluorescence is measured, and from the resonant frequency the local magnetic field strength can be inferred.

Magnetic field imaging was performed using a custom inverted wide-field microscope. Optical excitation at 532 nm was used to excite the nitrogen-vacancy (NV) centers in the diamond magnetic sensing chip. The resulting NV fluorescence was captured, filtered, and imaged onto a sensitive scientific complementary metal-oxide-semiconductor (sCMOS) camera. A microwave antenna was used to apply the spin control sequences required to infer local magnetic fields via optically detected magnetic resonance (ODMR), as shown in Fig. 1*A*. Thin tissue samples from the lagena and basilar papilla in the cochlear duct of adult pigeons were prepared, as illustrated in Fig. 1*B*. The samples were embedded in epoxy resin, and regions of interest were microtomed to a thickness of 500 nm (*Materials and Methods*). The sections were then transferred to the diamond on a drop of bidistilled water and air dried under a microscope.

The NV defect in diamond consists of a substitutional nitrogen atom with an adjacent lattice vacancy aligned along one of four diamond crystallographic axes. Under 532-nm optical excitation this defect can be initialized into the ∣0〉 spin sublevel. The spin state can be read out optically from the fluorescence intensity difference between the ∣0〉 and ∣±1〉 sublevels as illustrated in Fig. 1*C*. The positions of these energy levels can be determined by monitoring the NV fluorescence intensity while sweeping a microwave field through the respective ∣0〉 to ∣±1〉 transitions. The resulting ODMR spectrum can then be acquired from each imaging pixel of the quantum magnetic microscope. Static magnetic fields aligned with an NV axis Zeeman split the ∣±1〉 and ∣−1〉 states with a gyromagnetic ratio of *γ* = 2.8 MHz G^– 1^. The Zeeman splitting can, therefore, be used to report the local magnetic field projected along the NV axis at each imaging pixel, as shown in Fig. 1*D*. Here an ODMR protocol (14) is applied to image the stray static magnetic fields from individual cuticulosomes under a range of applied magnetic field strengths.

### Stray Magnetic Field Imaging

With the samples mounted, a series of ODMR images were recorded to screen for magnetic signals present in the hair cells in the lagena and basilar papilla. The diamond sensing chips were able to hold three tissue sections allowing for 300 individual hair cells from the lagena or 80 individual hair cells from the basilar papilla to be scanned on a single sensor chip. The field of view of the microscope was 150 μm${}^{2}$, and the magnetic image resolution was 450 ±50 nm. The local magnetic field projection along the NV axis was determined by monitoring the ODMR peak separation (15). The initial screening process involves magnetizing the cuticulosomes via application of a 1,400 G applied field. The initial acquisition times for each 150 μm${}^{2}$ field of view was between 30 and 60 min. Any magnetic features observed in the initial screen were then targeted for detailed analysis.

**Fig. 2:**
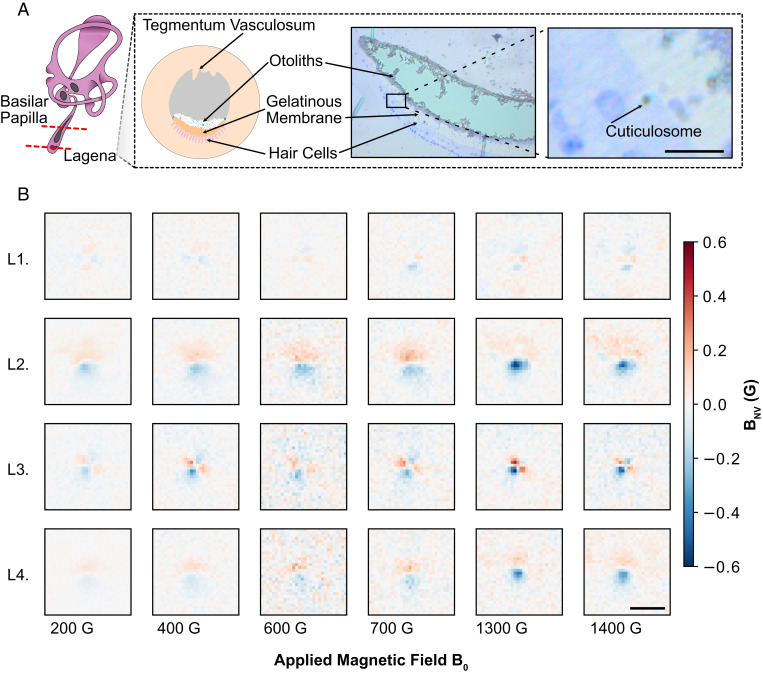
Cuticulosome measurements and the anatomical origins of the cuticulosomes investigated. (*A*) Schematics and bright-field images showing the orientation of the sections taken, the location of the lagena within the inner ear, and the components of interest within the sections. The second bright-field image is an enlargement of the region indicated by the black rectangle in the first. The black arrow indicates the location of a cuticulosome within the tissue sample. (Scale bar, 5 μm.) (*B*) ODMR images showing the stray magnetic field imaged below cuticulosomes found in the lagena. The applied magnetic field increases from left to right from 200 to 1,400 G with each row representing a different particle. Cuticulosome L1 is the same cuticulosome shown in *A*. (Scale bar, 5 μm.)

**Fig. 3:**
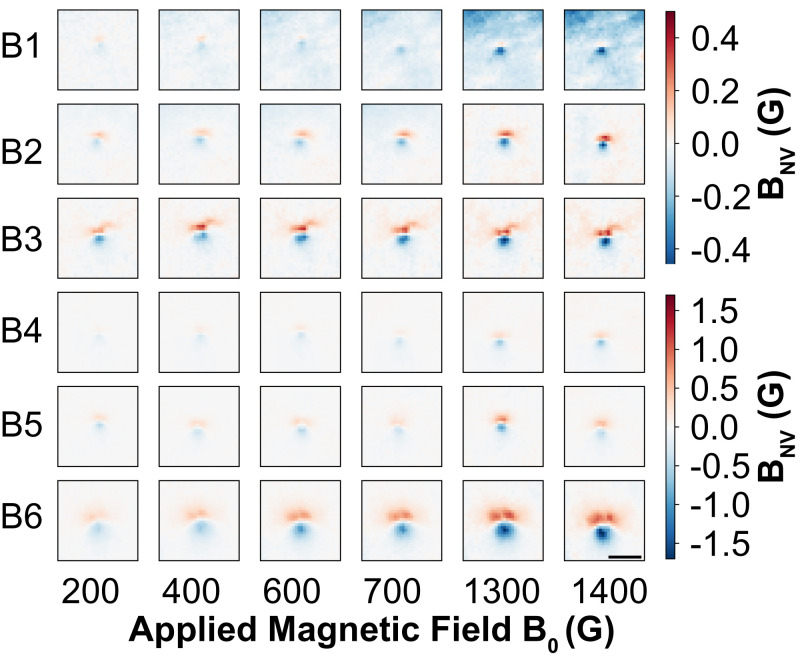
ODMR images showing the stray magnetic field imaged below cuticulosomes found in the basilar papilla. The applied magnetic field increases from left to right from 200 to 1,400 G with each row representing a different particle. (Scale bar, 5 μm.)

To provide insight into the magnetic susceptibility of iron cuticulosomes, magnetic images were taken under a range of applied fields, *B*_0_, ranging from 100 to 1,900 G. Cell surveys of the lagena and basilar papilla, as shown in Fig. 2*A*, identified approximately four and six iron cuticulosomes from 300 and 80 hair cells, respectively. The resulting stray magnetic field profiles from the individual cuticulosomes present in the lagena and basilar papilla are shown in Figs. 2*B* and 3, respectively. The image acquisition details are outlined in *Materials and Methods*. Differences in the observed stray magnetic spatial profiles can be accounted for by the height between the NV sensing layer and the iron cuticulosomes in the tissue and are discussed further in *SI Appendix*.

**Fig. 4:**
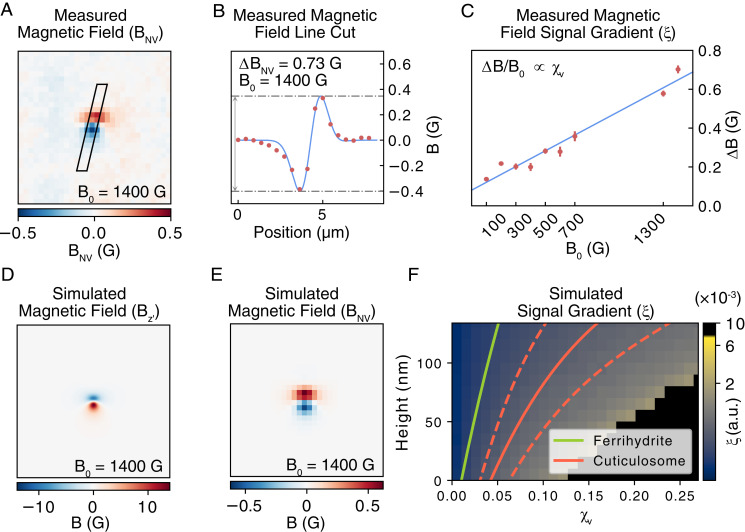
Calculation of the magnetic susceptibility of a cuticulosome via the comparison of stray magnetic field images with a detailed theoretical model. (*A*) Representative image of the stray magnetic field as inferred through the analysis of ODMR images. The axis width is 13 μm. (*B*) Line cut through the center of the stray magnetic field measured in *A*. The peak to peak size of the double Gaussian fits to the data provides a metric for of the strength of the signal. (*C*) Plot of the strength of the signal as a function of the applied magnetic field, B_0_. Error bars are calculated as the SD of the background. (*D*) Simulated stray magnetic field. The axis width is 13 μm. (*E*) Simulated effective magnetic field accounting for the integrated NV response and the optical diffraction limit. The axis width is 13 μm. (*F*) Simulation presenting the gradient of the signal strength as a function of applied field for a range of particle heights and magnetic susceptibilities. The green line represents the response produced by a sphere of ferrihydrite of the same diameter. The solid red line represents the points which result in the same NV response as the average of the experimentally measured NV responses. The red dashed lines represent the average experimentally measured NV response plus or minus 1 SD. The blacked out region of the map represents regions where an increased microwave frequency sweep range would be required to extract the resonant frequencies from all pixels of the simulated ODMR images.

To quantify the stray magnetic field strengths, a line cut was taken to infer the peak to peak magnitude of the measured field $\Delta B_{NV}$ as shown in Fig. 4 *A* and *B*. This procedure was repeated over the range of applied fields to study the relationship between the stray field strength, $\Delta B_{NV}$, and the applied field, *B*_0_, as shown in Fig. 4*C*. The gradient of the stray field vs. the applied field will here be referred to as *ξ* and is proportional to the magnetic susceptibility. The average values of *ξ* determined for cuticulosomes in the lagena and basilar papilla were $(3.5\pm 1.5)\times 10^{-4}$ and $(2.3\pm 0.9)\times 10^{-4},$ respectively. The value for *ξ* measured for each individual particle is documented in *SI Appendix*. To gain further insight into the magnetic images an analytical model is developed and applied to extract estimates of the magnetic susceptibility of the cuticulosomes as described in the section below.

### Stray Magnetic Field Modeling

The magnetic field model for the iron cuticulosomes takes into account the externally applied magnetic field, magnetic susceptibility, easy axis orientation, and cuticulosome geometry. The cuticulosomes are modeled as a large number of magnetic dipoles, producing a field,

[1] $$B_{\text{c}}(r)=\frac{\chi_{v}VB_{0}}{4\pi }\left(\frac{3r(\hat{m}\cdot r)}{r^{5}}-\frac{\hat{m}}{r^{3}}\right),$$

where *B*_0_ is the applied magnetic field, *χ_v_* is the volume magnetic susceptibility of the cuticulosome, *V* is the volume of the cuticulosome, and $\hat{m}$ is the direction of magnetization of the cuticulosome. The Zeeman splitting induced from an individual NV center at a position **r** relative to the center of the cuticulosome is calculated from the projection of $B_{\text{c}}$ along the axis of that NV center and will be referred to as $B_{z\prime }$ from here on.

Fig. 4*D* shows a simulation of $B_{z\prime }$ for the measured cuticulosome in Fig. 4*A*. The simulation was performed with a volume susceptibility of 0.053, a cuticulosome diameter of 365 nm, and a height of 207.5 nm, with an easy axis aligned with the applied magnetic field (1,400 G). Crystalline magnetic materials have a direction in which an applied magnetic field will more readily magnetize the material, with that direction being known as the easy axis of the material (16). The presence of an easy axis occurs due to the combination of magnetocrystalline effects, shape anisotropy, and surface effects creating energetically preferred spin alignments. The model assumes a cuticulosome geometry with an aspect ratio of 1. This is informed by electron microscopy studies conducted by Lauwers et al. (6). The image presented in Fig. 4*D* was generated for the case where the easy axis and applied field are aligned. A packing ratio of 0.7 was assumed for the efficiency of the infilling of the cuticulosome volume with magnetic material (17). The resolution of the image is set to be 5 nm.

The theoretical model developed here also considers physics behind the quantum magnetic imaging system to produce an effective stray magnetic field which accounts for the NV dynamic range, implant depth, cuticulosome height, and optical imaging resolution (18). See *SI Appendix* for more details. The resulting simulated effective stray magnetic field, as shown in Fig. 4*E*, can then be compared with the measured magnetic field profiles. There is excellent qualitative agreement between the two profiles. An upper bound on the variation that would result from magnetic anisotropy was determined by simulating the response from a large number cuticulosomes with uniaxial magnetization. Easy axis directions were generated using a Monte Carlo simulation. Apart from the easy axis direction, all parameters of the simulations were identical to each other and to the cuticulosome simulated in Fig. 4 *D* and *E*. By comparing the SD of the stray magnetic field amplitudes to their mean for 300 instances of the Monte Carlo simulation, a relative error in *ξ* of 0.25 was determined.

Leveraging the theoretical model allows us to place bounds on the magnetic susceptibility based on the experimental data. Due to the number of free parameters in the theoretical model it is not possible to calculate the magnetic susceptibility directly as a function of *ξ*. However, by repeating the simulations over the physically realistic range of cuticulosome radii and heights it is possible to place bounds on the susceptibility of individual particles. *ξ* is calculated for each parameter set, producing a finite range of magnetic susceptibilities capable of reproducing the experimental data. This method is presented in Fig. 4*F* where a representative particle diameter of 365 nm is chosen, in line with an analysis of cuticulosomes performed via electron microscopy (6), and where the cuticulosome height is swept over the tissue sample thickness. Contour lines depicting the combinations of height, magnetic susceptibility, magnetic anisotropy, and radius which are consistent with experimental data are drawn to impose limits on the magnetic susceptibility of cuticulosomes. The lower and upper bounds on the magnetic susceptibilities inferred through these simulations are 0.029 and 0.22, respectively. These susceptibilities are an order of magnitude less than those reported for magnetite (19), suggesting that the iron cuticulosomes are composed of composite iron biominerals with magnetic susceptibilities more consistent with ferrihydrite. These values can be compared to the magnetic susceptibilities of known iron oxide species as listed in Table 1.

**Table 1:** Magnetic susceptibility ranges and mass densities of a variety of iron oxide species

Species	*χ_m_*	*ρ*	*χ_v_*
Ferrihydrite	0.25* to 2.1^†,‡^	1,200*	$(3.8-32)\times 10^{-4}$
Magnetite	230 to 950*	5,180*	1.5 to 6.2
Maghemite	180 to 530^§^	4,860^¶^	1.1 to 3.2
Hematite	0.10 to 3.0^§^	5,260^¶^	$(6.6-200)\times 10^{-4}$
Goethite	0.37 to 4.8^§^	3,300 to 4,300^¶^	$(1.5-26)\times 10^{-3}$
Cuticulosomes	27 to 200	1,200 to 5,260	0.029 to 0.22

*χ_m_* is presented in units A m${}^{2}$ kg^– 1^ T^– 1^, *ρ* is presented in units kg m^– 3^, and *χ_v_* is dimensionless.

* Data are provided from ref. 19.

^†^ Data are provided from ref. 34.

^‡^ Data are provided from ref. 35.

^§^ Data are provided from ref. 33.

^¶^ Data are provided from ref. 36.

To understand if the magnetic susceptibilities measured are consistent with the ferrimagnetic particle–based model, the force required to mediate a magnetoreceptive response is compared to the force produced by cuticulosomes in geomagnetic fields. The force required for the gating of mechanoelectrical transduction channels is assumed to be comparable to the gating force measured in saccular hair cells of a bullfrog, which is $(2.9\pm 0.6)\times 10^{-13}$ N (20). The upper bounds of the forces capable of being exerted by cuticulosomes are calculated to determine the feasibility of cuticulosomes acting as mediators of magnetic field information. The calculations to determine the upper bounds are presented in *Materials and Methods* and produce a maximum value of $10^{-18}$ N which is five orders of magnitude too small to gate known mechanoelectrical channels. As such, the data obtained from the analysis do not support the proposal that cuticulosomes act as particle-based mediators of magnetic fields and complement theoretical treatments of the behavior of cuticulosomes in geomagnetic fields (19).

The results reported herein support the claim that cuticulosomes contain several iron oxide minerals (21). The magnetic susceptibilities measured are not consistent with either the modeling of cuticulosomes as solid spheres of magnetite or as solid spheres of ferrihydrite. Low-temperature magnetic studies on horse spleen ferritin have provided evidence of mixed mineral phases of ferrihydrite and magnetite/maghemite (22). The measurement of mixed mineral phases measured in horse spleen ferritin provides the motivation to further analyze the mixed mineral composition observed within cuticulosomes. Information regarding the mineral composition of cuticulosomes was gleaned using stray magnetic field imaging to quantify their magnetic susceptibilities. Additional information can be obtained by studying the superparamagnetism of cuticulosomes. This was undertaken through quantum relaxometry and is discussed in the next section.

### Quantum Relaxometry Imaging

The magnetic domains associated with superparamagetic materials are known to fluctuate with a frequency spectrum dependent on the volume of the magnetic material, anisotropy energy barrier, mineral composition, and temperature. For iron biominerals such as ferrihydrite, this frequency spectrum can range between several hundred MHz and tens of GHz (14). These magnetic fluctuations can act as magnetic noise sources when brought sufficiently close (typically < 100 nm) to NV centers in diamond. By using the same wide-field microscope and monitoring the spin relaxation rate ($1/T_{1}$) of the NV centers across the full field of view (*Materials and Methods*), we can detect and map the fluctuating magnetic fields signals directly, in a technique termed quantum relaxation microscopy (QRM) (23–25). For completeness we applied QRM to image each of the iron cuticulosomes identified in Figs. 2 and 3; see *SI Appendix* for more information.

Two out of the 10 cuticulosomes studied show a clear change in *T*_1_ rate. Further work is required to understand whether the low percentage of cuticulosomes reporting superparamagnetic domains is statistically significant or whether the sample preparation and thickness limits *T*_1_ detection events. Regardless of the statistical significance of the number of detection events, the successful detection of fluctuating magnetic signals from individual cuticulosomes indicates that the particles are composed of both superparamagnetic and ferrimagnetic domains. It is likely that cuticulosomes may contain magnetic granules with a range of sizes and morphologies with some particles acting as superparamagnets while others act as ferrimagnets. A similar effect has been observed in clusters of synthetic maghemite nanoparticles with diameters in the 5 to 50 nm range (26). Our results highlight the nonuniformity in the magnetic properties of these materials prompting further investigation into the physiological significance of these organelles.

## Conclusion

Here we applied wide-field quantum magnetic microscopy in two separate magnetic imaging modalities to characterize the magnetic properties of individual cuticulosomes in vestibular and acoustic hair cells of pigeons. Magnetic images were captured from 500-nm-thick sections of tissue taken from two distinct locations within the cochlear duct. ODMR microscopy was used to image the stray magnetic field from individual cuticulosomes. The stray magnetic field response to an applied magnetic field was compared to a detailed analytical model to determine the magnetic susceptibility of cuticulosomes. The magnetic susceptibilities measured to be 0.12 ±0.05 indicate that cuticulosomes do not have sufficient magnetization to act as a particle-based magnetoreceptor. This does not discount the other proposed physiological roles of cuticulosomes, namely, that they act as mechanical stabilizers of stereocilia or as iron stores. The magnetic susceptibility of cuticulosomes lies between the magnetic susceptibility of ferrihydrite and magnetite, motivating further study of their mineral components. With this in mind, quantum relaxometry was applied to probe the superparamagnetic components of these iron organelles. Two of the 10 organelles studied exhibited superparamagnetic properties suggesting the presence of multiple iron oxide minerals within individual cuticulosomes. Importantly, the magnetic images can be correlated to specific anatomical locations, mitigating the risk of false positives from magnetic contamination. This demonstrates the utility of quantum magnetic imaging as a means to screen for similarly small-scale magnetoreceptor candidates within biological samples. Given the simplicity of the sample preparation and imaging technique, future work will focus on applying this technology to a host of biological systems with preidentified magnetic particle–based magnetoreceptor candidates to further understand the role biogenic iron plays in animal physiology.

## Materials and Methods

### Sample Preparation

Adult pigeons (Austria cohort) were killed by CO_2_ asphyxiation and immediately intracardially perfused with 40 ^∘^C 0.9% NaCl supplemented with 20 U/L heparin followed by a mixture of ice-cold 2% paraformaldehyde (PFA) and 2.5% glutaraldehyde (GA) in phosphate-buffered saline (PBS, pH 7.4). The inner ear was dissected and postfixed in 2%PFA/2.5%GA at 4 ^∘^C overnight. To facilitate the penetration of the fixatives, the oval window and the semicircular canals were cut open. To limit contamination, all preparations were made with acid-washed (1% HCl) iron-free titanium and ceramic tools. Following postfixation the samples were washed in PBS for 1 h, and the cochlear duct containing the lagena and basilar papilla was removed. Next, the tissue was washed with Soerensen 0.1 M phosphate buffer (PB), dehydrated, and embedded in epoxy resin. After polymerization, the blocks were trimmed, and 500-nm sections at the level of the basilar papilla were prepared using a diamond knife on a Leica UCT ultramicrotome. Single sections were transferred onto the diamond chip in a drop of bidistilled water using a loop. They were air-dried under continuous visual inspection at a stereomicroscope to ensure flat mounting. All experiments were performed in accordance with an existing ethical framework (GZ:214635/2015/20) granted by the City of Vienna (Magistratsabteilung 58).

### Quantum Magnetic Imaging

The substrates used in this experiment were 〈100〉 oriented chemical vapor deposition grown diamond from Delaware Diamond Knives. Nitrogen vacancy centers were created by implanting ${}^{15}$ N with a dose of $1\times 10^{13}$ cm^– 2^ at 4 keV and an implant angle of 7^∘^ before annealing at $1,000{\;}^{{}^{\circ}}$C. After annealing, the spatial density of NV centers in the diamond is approximately $1\times 10^{11}$ cm^– 2^ (27). Of the NVs on the surface, 75 to 80% exist in the negatively charged state used for magnetic imaging (28). The external magnetic field used to magnetize the cuticulosomes was provided by neodymium magnets positioned using Scientifica motorized micromanipulators or a Physik Instruments motorized XYZ stage. A microwave signal is generated from an Agilent MXG N5183A analog signal generator with the signal then being amplified and delivered to the diamond sample by gold omega-shaped antennas printed on glass coverslips. Microwave switching is provided by a transistor–transistor logic–driven Mini-Circuits microwave switch (ZASWA-2-50DR+). Laser excitation is provided by a Lighthouse Photonics Sprout-G with laser switching provided by acousto-optic modulators (AOMs) with the radio frequency (RF) to the AOM being produced by a MOGLabs Agile RF synthesizer. The fluorescence produced by the nitrogen vacancy layer was separated from the excitation using a dicroic mirror before being collected by an Andor Neo 5.5 sCMOS camera or an Andor Zyla 5.5 sCMOS camera. The pulses required for the control of the AOM, microwave switch, and camera shutter are produced using a SpinCore Pulse blaster ESR-Pro-500. Nikon oil immersion objectives with a numerical aperture of 1.30 were used for these experiments. The laser power at the back of the objective was ≈250 mW, which corresponds to a power density of 1.7 kW mm^– 2^.

### Derivations of Magnetic Forces from Cuticulosome Susceptibilities

The magnetic forces due to several different mechanisms were calculated to determine the plausibility of cuticulosomes acting as mediators of geomagnetic field information. The torque resulting from misalignments between the geomagnetic field and the magnetic moment of cuticulosomes, the magnetic force acting between cuticulosomes in close proximity, and the magnetic force resulting from the interaction between the magnetic moment of cuticulosomes and the spatial gradient of the geomagnetic field were calculated. These calculations resulted in forces at least five orders of magnitude too small to gate known mechanoelectrical channels.

The torque acting on a cuticulosome is calculated following the method outlined by Winklhofer and Kirschvink (30). Torques can occur in magnetic particles with induced magnetizations. Magnetic anisotropy can produce a misalignment between the applied and induced fields. The torque is calculated as the cross product of the applied field and the net magnetization of the magnetic particle. Expanding the cross product allows for the determination of the three components of the torque as

[2] $$\vec{\tau }=\frac{1}{2}\left(\begin{array}{l} (\chi_{b}\prime -\chi_{c}\prime )\sin\;\left(\phi \right)\sin\;\left(2\theta \right)\\ (\chi_{c}\prime -\chi_{a}\prime )\cos\;\left(\phi \right)\sin\;\left(2\theta \right)\\ (\chi_{a}\prime -\chi_{b}\prime )\sin\;\left(2\phi \right){\sin\;}^{2}\left(\theta \right) \end{array}\right)B^{2}V/\mu_{0},$$

where *θ* and ϕ are the longitudinal and azimuthal angles, respectively, between the easy axis of the magnetic particle and the applied field; *B* is the applied field; and $\chi_{a}\prime ,\;\chi_{b}\prime$, and $\chi_{c}\prime$ are the magnetic susceptibilities along the principal axes of the magnetic particle. Applying the above equations to a cuticulosome with a volume of $2\times 10^{-19}$ m${}^{3}$, $\chi_{a}\prime =\chi_{b}\prime =0.18$, and $\chi_{c}\prime =0.19$ in geomagnetic fields produces a torque of $2.6\times 10^{-25}$ Nm and a corresponding force at the surface of the particle of $1.4\times 10^{-18}$ N.

The force exerted by two cuticulosomes in close proximity to one another was also considered, following the equations derived by Davila et al. (31). This force increases as the distance between particles decreases, reaching a value of $10^{-19}$ N when the distance between the particles is twice their radius.

Magnetic forces are also exerted on cuticulosomes when the applied magnetic field has a spatial gradient at the position of the cuticulosome. This is calculated as the gradient of the magnetic potential energy, $U=\frac{1}{2}mB_{\mathrm{geo}}$, where *m* is the magnetization of the cuticulosome and *B_geo_* is the geomagnetic field (2). The magnetization can be calculated as the product of the magnetic susceptibility of the cuticulosome. Assuming a magnetic susceptibility, *χ_v_*, of 0.053, a geomagnetic field of 0.5 G, a cuticulosome radius of 365 nm, and a geomagnetic field gradient of $1\times 10^{-6}$ G, a magnetic force of $1\times 10^{-33}$ N (32) is estimated.

### Pulsed ODMR Acquisition

A pulsed-ODMR control sequence is applied to acquire ODMR spectra. The fluorescence is collected and binned with a coarseness of 2  ×  2 by the camera. For each microwave frequency in the spectrum, an optical pulse was applied to spin-polarize the NVs into the ∣0〉 sublevel. A microwave pulse of a duration set to the *π* time of the NV centers was applied, where the *π* time is the duration which maximizes the efficiency of the spin transition when the microwave frequency is on resonance. A reference is also taken for each data point which consists of the same pulse sequence excluding the microwave pulse. The resulting data point for each microwave frequency is the ratio of the signal to the reference. The acquisition procedure is implemented using a custom LabVIEW program.

### Quantum Relaxometry Acquisition

Quantum relaxometric microscopy maps the presence of magnetic noise by measuring the time taken for spin-polarized NVs to lose their polarization. The presence of magnetic noise with frequency spectra overlapping the spin transition frequencies of the NVs causes a more rapid loss of spin polarization or an increase in the *T*_1_ rate. The control sequence applied to acquire a data point of a *T*_1_ spectrum involves first optically polarizing the NV layer into the ∣0〉 state before allowing the spin state to evolve in the dark for some time, *τ*, before the final spin polarization is measured. A reference measurement for each *τ* time is also taken, in which a microwave *π* pulse is applied prior to readout. The reference mitigates against unwanted effects including background light. Each data point in the resulting *T*_1_ spectrum is the ratio of the signal to the reference. The *τ* times are logarithmically spaced to maximize the collection of information about the NV decay rate, as collection of data points where *τ* is large is more time consuming than the collection of data points where *τ* is small. *T*_1_ spectra are collected across the full field of view of the sCMOS sensor, with the data binned with a coarseness of 2  ×  2.

### ODMR Data Analysis

Custom MATLAB and Python programs were written to extract the resonances of interest from the collected data and extract magnetic information from the resonances. The analysis program bins the collected data with a coarseness of 2  ×  2. The locations of resonant frequencies in ODMR spectra at each binned pixel are extracted by fitting a Lorentzian function,

[3] $$I(\omega )=1-\text{C}\frac{\Gamma^{2}}{\Gamma^{2}+{\left(\omega -\omega_{0}\right)}^{2}},$$

where I(ω) is the normalized fluorescence intensity as a function of the applied microwave frequency, C is the contrast of the ODMR peak, Γ is a factor to specify the width of the Lorentzian fit, and *ω*_0_ is the resonance frequency, to the experimental data at each binned pixel. To maximize the sensitivity the microwave driving power which maximizes the ratio of C to Γ is sought and applied.

A Gaussian filter is applied to the image where the Gaussian kernel has a SD of 50 pixels to create a coarse background. The pixels overlapping the signal are replaced by pixels in the corresponding coarse background image to create an image with the signal removed, which enables the production of a background image which is less impacted by the presence of the signal. The background is then calculated by applying a Gaussian filter to the preprocessed image. The SD of the Gaussian kernel is set to 25 pixels. The signal was then obtained by subtracting the background from the unaltered image.

The metric for the stray magnetic field size employed here was produced by taking a line cut of a width of three pixels through the stray magnetic field signal maximum. A double Gaussian function was then fit to the line cut for each image, with the peak to peak magnitude of this fit providing a numerical indication of the stray magnetic field strength.

### Quantum Relaxometry Data Analysis

The same analysis programs are able to extract the *T*_1_ rates from *T*_1_ spectra. The analysis program again bins the data with a coarseness of 2  ×  2. The stretched exponential function,

[4] $$I(\tau )=1+\text{C}\exp\;\left(-{\left(\frac{\tau }{T_{1}}\right)}^{p}\right)-\text{C},$$

where *I*(*τ*) is the fluorescence intensity as a function of the dark time, C is the contrast, *T*_1_ is the *T*_1_ time, and *p* is the stretching exponent, is fit to the data at each binned pixel. The stretching exponent is included to account for effects such as the range of NV depths in the NV layer. A decrease in the value of *T*_1_ is expected to be observed in the vicinity of superparamagnetic material.

## Supplementary Material

Supplementary File

## Data Availability

Raw data have been deposited in Zenodo (https://doi.org/10.5281/zenodo.5610212). All other data are included in the article and/or *SI Appendix*.
