# Supplementary material for: Quantum magnetic imaging of iron organelles within the pigeon cochlea
Source: Proc Natl Acad Sci U S A. 2021 Nov 15;118(47):e2112749118. doi: 10.1073/pnas.2112749118 (PMC8617482; doi:10.1073/pnas.2112749118)
Supplement: Supplementary File [file pnas.2112749118.sapp.pdf]

Supplementary Information for

Quantum Magnetic Imaging of Iron Organelles Within the Pigeon Cochlea

Robert W. de Gille, Julia M. McCoey, Liam T. Hall, Jean-Philippe Tetienne, E. Pascal Malkemper, David A. Keays, Lloyd C. L. Hollenberg and David A. Simpson

David A. Simpson.
E-mail: simd@unimelb.edu.au

This PDF file includes:

Supplementary text
Figs. S1 to S7
Table S1
SI References

Supporting Information Text

ODMR Pulse Sequence

Nitrogen vacancy centers in diamond are used as magnetic field sensors by exploiting the Zeeman effect. Applying a magnetic field along one of the four diamond crystallographic axes splits the $|+1\rangle$ and $|-1\rangle$ ground state spin sub-levels of aligned NV centers with a gyromagnetic ratio of $2.8\,\mathrm{MHz\,G^{-1}}$. The fluorescence intensity difference between the $|0\rangle$ and $|\pm1\rangle$ spin states provides a convenient optical method for spin state readout. Optical excitation also initialises NV centers into the $|0\rangle$ spin sub-level which, in conjunction with the application of resonant microwave fields, enables coherent spin state control of NV ensembles. Optically Detected Magnetic Resonance (ODMR) is a protocol which leverages spin intialisation, manipulation and readout to determine the transition energies between the $|0\rangle$ and $|\pm1\rangle$ spin states. A map of the local magnetic field can be formed by gathering the ODMR spectrum at each imaging pixel.

Continuous Wave ODMR (CW-ODMR) and Pulsed ODMR are two protocols ubiquitous in NV magnetometry. CW-ODMR is implemented by applying optical and microwave pulses throughout the measurement. Under the pulsed ODMR measurement scheme, the NV centers are first initialised into the $|0\rangle$ state using an optical polarisation pulse. The length of the microwave pulse is tuned to be the NV π-time, which is the microwave pulse duration which maximises the conversion between the spin sub-levels. An optical readout pulse is applied to determine the final spin state populations. This measurement is taken along with a reference measurement which omits the microwave pulse. Each data point of the ODMR spectrum is the ratio of the signal divided by the reference. The microwave power is chosen such that at a representative pixel the ratio of the contrast to the width is maximised. Pulsed ODMR achieves an improved sensitivity over CW-ODMR, as the reduced laser pulse duration mitigates sensitivity losses due to re-pumping of the NVs throughout measurement (1). The pulsed ODMR protocol has the additional benefit of reducing the optical damage suffered by the sample due to laser exposure. The Pulsed ODMR scheme was chosen for use in this work due to the aforementioned benefits. The pulse sequence to acquire two data points on an ODMR spectrum is presented in Fig. S1A. An example spectrum acquired using this pulse sequence is presented Fig. S1B.

Rabi Pulse Sequence

Rabi oscillations are used to determine the average π-time of the NVs across the full field of view. The protocol initialises the NV centers in the $|0\rangle$ state and drives continuously at one of the resonant frequencies for some time τ before reading out the final fluorescence. An oscillation in the final fluorescence intensity can be observed as the NV centers are driven between the bright and dark spin sub-levels, with the first minimum of this oscillation occurring where τ is the π-time. The pulse sequence used for this measurement and an example rabi oscillation are presented in Fig. S2.

Spin lattice relaxation (T_1) Pulse Sequence

Additional information regarding the magnetic properties of cuticulosomes are observed through the use of quantum relaxometric imaging. Quantum relaxometric imaging probes magnetic fluctuations by monitoring the spin lattice relaxation times (T_1) of the NVs. Magnetic fluctuations at frequencies near the NV transition frequency of 2.87 GHz decrease the spin lattice relaxation time of the NVs. The pulse sequence implemented to measure the T_1 time consists of an optical excitation pulse and an optical readout pulse separated by a dark evolution time, τ. A reference measurement is also taken, where the spin population is inverted by a microwave π-pulse before the final spin state is read out. The T_1 decay is obtained by taking the ratio of the signal to reference. The dark evolution times, or τ times, are spaced logarithmically to optimise the data acquisition rate. The pulse sequence used to implement quantum relaxometry is presented in Fig. S3 along with an example T_1 decay curve. The T_1 decay curves are fit using a stretched exponential function of form $I(t) = 1 + \mathscr{C}\exp[-(t/T_1)^p] - \mathscr{C}$, where $\mathscr{C}$ is the contrast, T_1 is the spin lattice relaxation time and p is the power of the stretched exponential (typically between 0.7 and 1).

Quantum Relaxometry Imaging Results

Two out of the ten cuticulosomes surveyed displayed a measurable T_1 signal. The T_1 rate images ($1/T_1$) for the two measurable particles are presented in Fig. S4 along with the corresponding SEM or optical brightfield image of the same region. The change in T_1 rate verifies that fluctuating magnetic field contributions from individual iron cuticulosomes are present. The T_1 imaging method probes an extremely localised ($< 100\,\mathrm{nm}$) volume. The 500 nm slice thickness may have resulted in a standoff between the cuticulosome and NVs $> 100\,\mathrm{nm}$ preventing a measurable T1 signal from being obtained. Future work on thinner sections will explore if a greater percentage of cuticulosomes exhibit fluctuating magnetic field components. The presence of fluctuating magnetic fields is not surprising given the ODMR image analysis indicates that the magnetisation of individual cuticulosomes is within an order of magnitude of superparamagnetic ferrihydrite.

Full Stray Field Results. Fig. S5 shows the measured field magnitude as a function of the applied magnetic field for the ten cuticulomsomes studied in this work. The gradient of the line of best fit for each particle, ξ, is presented in Table S1.

It is important to note that the measured stray magnetic fields profiles differ from that of a traditional dipole field. The difference arises when the source of the magnetic field is in close proximity to the NV layer (2). A combination of the strong magnetic field gradients across the area of the imaging pixel and the finite magnetic field sensitivity range of the NV defects causes a reduction of ODMR contrast and broadening of the ODMR peaks. This is due to the varying magnetic fields at the positions of different NV centers within the same imaging pixel. The variation in the spatial profiles of the stray magnetic

fields can be attributed to the relationship between the proximity effect and the height between the core of the cuticulosome and the NV sensing layer. In the section below, we establish an analytical model which accounts for these effects.

Analytical Model

To determine the response of the NV sensing layer to the magnetic field produced by an individual cuticulosome, we model the magnetic field from the cuticulosome as a 3D magnetic dipole field given by

$$\mathbf{B}_c(\mathbf{r}) = \frac{\chi_v V B_0}{4\pi}\left(\frac{3\mathbf{r}(\hat{\mathbf{m}}\cdot\mathbf{r})}{r^5} - \frac{\hat{\mathbf{m}}}{r^3}\right), \qquad [1]$$

where B_0 is the applied magnetic field, χ_v is the magnetic susceptibility of the cuticulosome, V is the volume of the cuticulosome and $\hat{\mathbf{m}}$ is the direction of magnetisation of the cuticulosome. The stray magnetic field from the cuticulosome was then used to emulate the integrated response of a large number of NVs in a thin sensing layer binned into appropriately sized pixels.

Multiplying the magnetization by the volume of the cuticulosome allows the magnetic moment from the particle to be determined. The magnetic field at any point in space can then be determined using the above equation for the magnetic field from a dipole. An example is presented in Fig. S6 for a cuticulosome with a diameter of 365 nm, a susceptibility of 2.6×10^{-3}, which is commensurate with the susceptibility of a cuticulosome composed solely of ferrihydrite, and a centroid height above the diamond imaging chip of 192.5 nm. The cuticulosome was magnetized with an applied magnetic field of 2000 G and the NV sensing layer depth was 10 nm beneath the diamond surface. The geometry is presented in Figs. S6A and B. Fig. S6A presents the laboratory coordinate system and Fig. S6B presents the coordinate system of the NV layer. The three components of the magnetic field at the NV layer are presented in Fig. S6(C-H), where Figs. S6(C-E) are the three components in the laboratory frame and Figs. S6(F-H) are the three components after transformation to the coordinate system of the NV layer.

The magnetic field projection along the axis of the NV is used to calculate the resonant frequency of each NV center in the sensing layer. This calculation is performed for a large number of nitrogen vacancy centers to accurately capture effects caused by magnetic field gradients within individual imaging pixels. An NV density of 1000 nitrogen vacancy centers per pixel was chosen. This density corresponds to a conversion of approximately 6% of the nitrogen atoms to NV centers in the real imaging devices and as a result will not underestimate the number of NV centers contributing to the signal (3). The x and y coordinates of each NV center placed within a pixel are chosen using a uniform random distribution. The z coordinates are chosen according to a normal distribution with a mean of the implantation depth and a standard deviation of half the implantation depth, which accounts for straggling which occurs in the nitrogen implantation process. The ODMR contrast and linewidth assumed in the simulations are 15% and 12 MHz respectively.

The simulated magnetic fields are then used to calculate the fluorescence intensity of each NV and for each microwave frequency within the microwave sweep range. Fig. S7A shows a set of 1000 ODMR spectra within a single imaging pixel directly below the hypothetical cuticulosome discussed. The contribution from each NV is then summed to simulate the ODMR spectrum which would be measured by each pixel of the imaging device. Fig. S7B illustrates a set of ODMR spectra where each spectrum relates to a particular pixel of the magnetic microscope and is calculated by summing the response of all the NV centers within the respective pixel. A Lorentzian function is fit to each spectrum and Gaussian blur is then applied to account for the optical diffraction in the imaging system. The fitting parameters extracted are then mapped for comparison with experimental results.

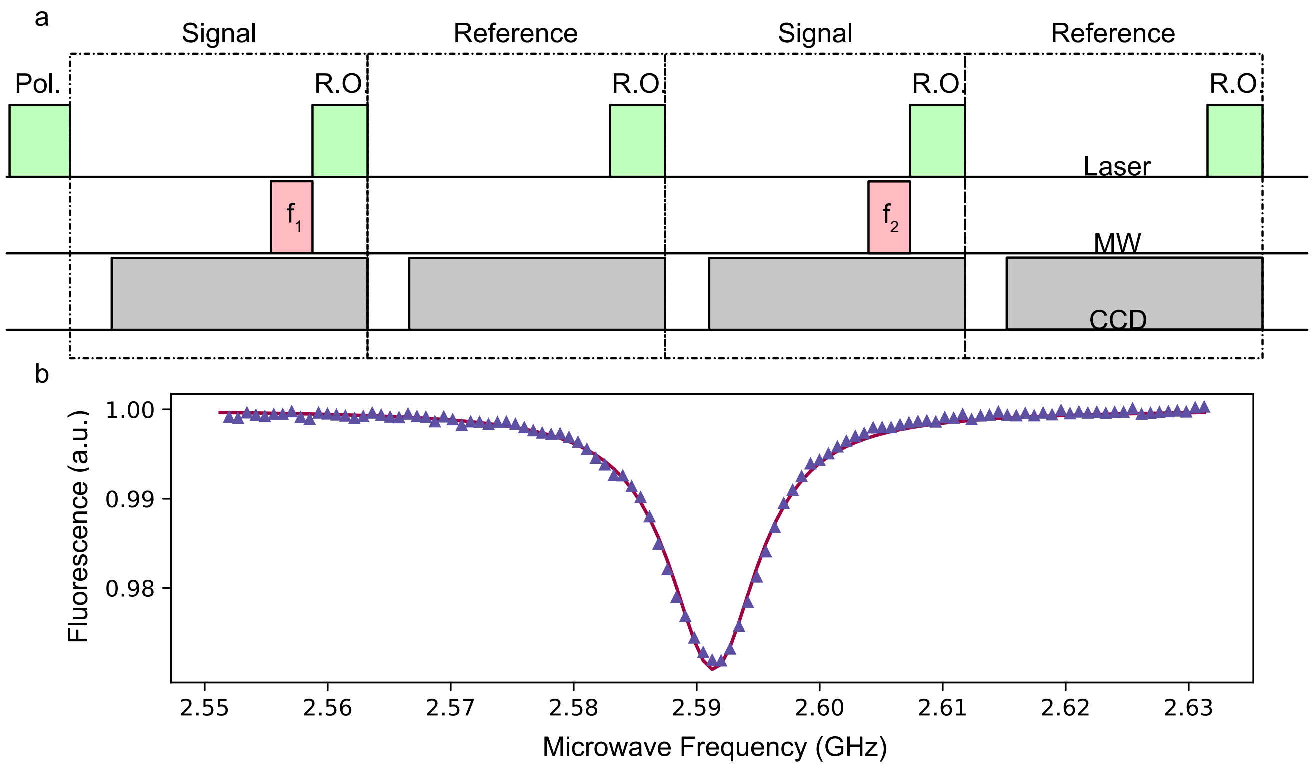

Fig. S1. Pulsed Optically Detected Magnetic Resonance overview. (A) Pulsed ODMR pulse sequence implemented to produce images of the static magnetic fields near cuticulosomes. (B) Example of an ODMR spectrum where each marker on the spectrum is calculated as the ratio of the signal to the reference. The maroon line is a Lorentzian fit to the data.

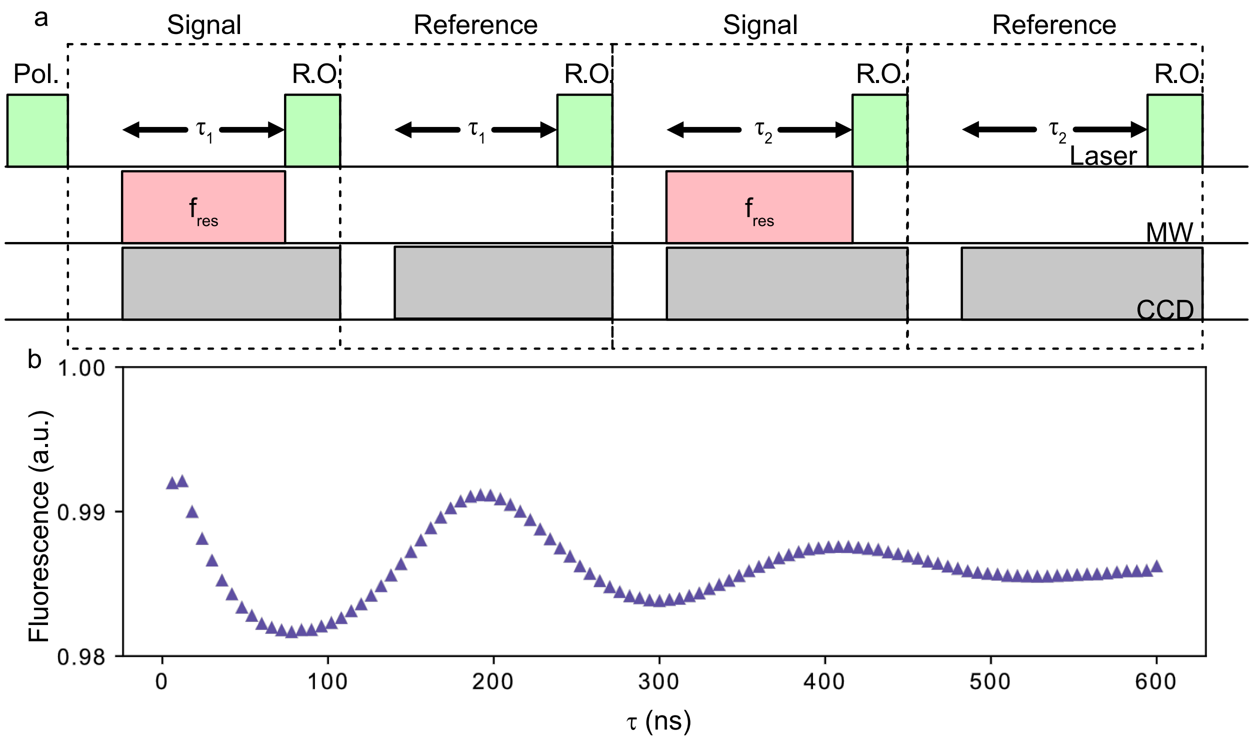

Fig. S2. Overview of the measurement of the NV π time. (A) Pulse sequence used to perform Rabi cycling for the measurement of the NV π-time. (B) Example Rabi spectrum. Each signal to reference ratio relates to a specific datum on the spectrum.

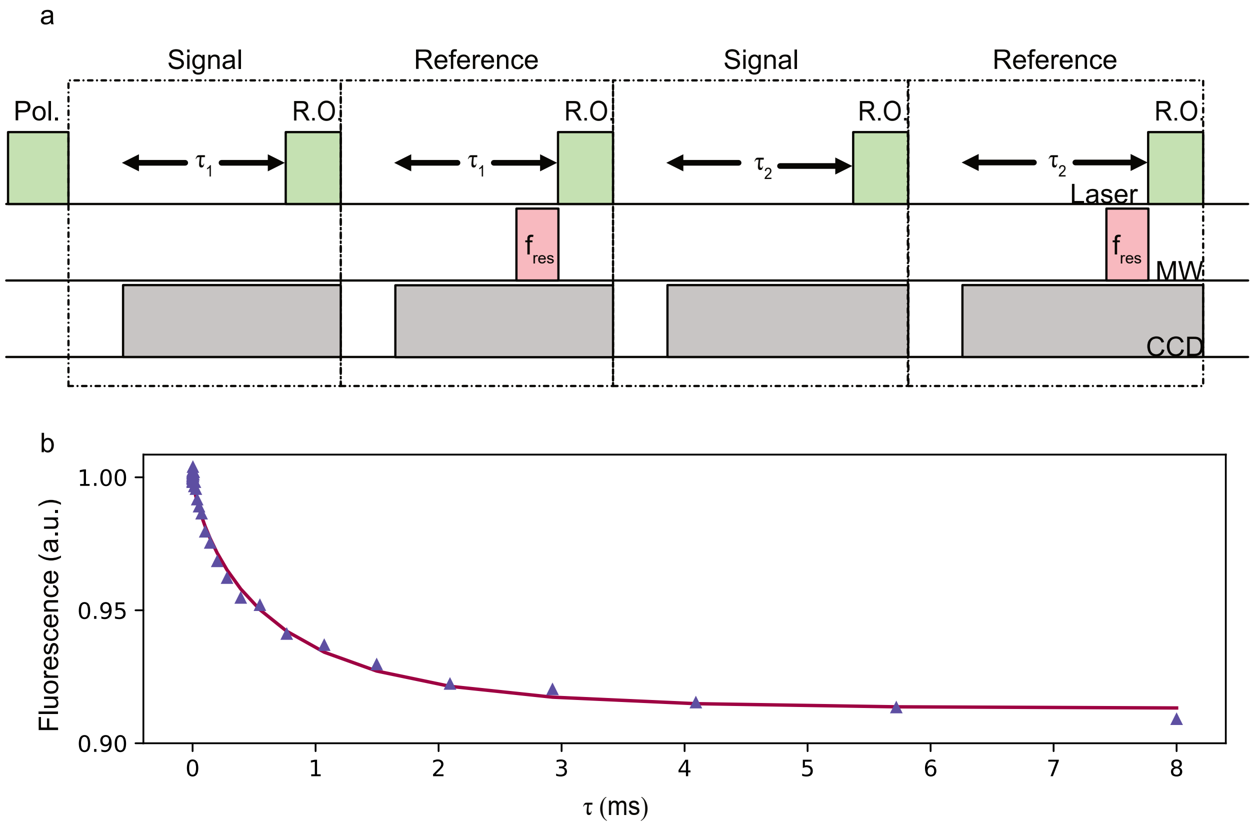

Fig. S3. Overview of the measurement of the NV T_1 time. (A) Pulse sequence used to probe the magnetic field fluctuations of the order of the splitting of the $|0\rangle$ and the $|\pm1\rangle$ spin sub-levels of the NV center. (B) Example T_1 spectrum. The markers were acquired using the pulse sequence depicted in A. The maroon line is a stretched exponential fit used to extract the T_1 time from the data.

 Robert W. de Gille, Julia M. McCoey, Liam T. Hall, Jean-Philippe Tetienne, E. Pascal Malkemper, David A. Keays, Lloyd C. L. Hollenberg and David A. Simpson

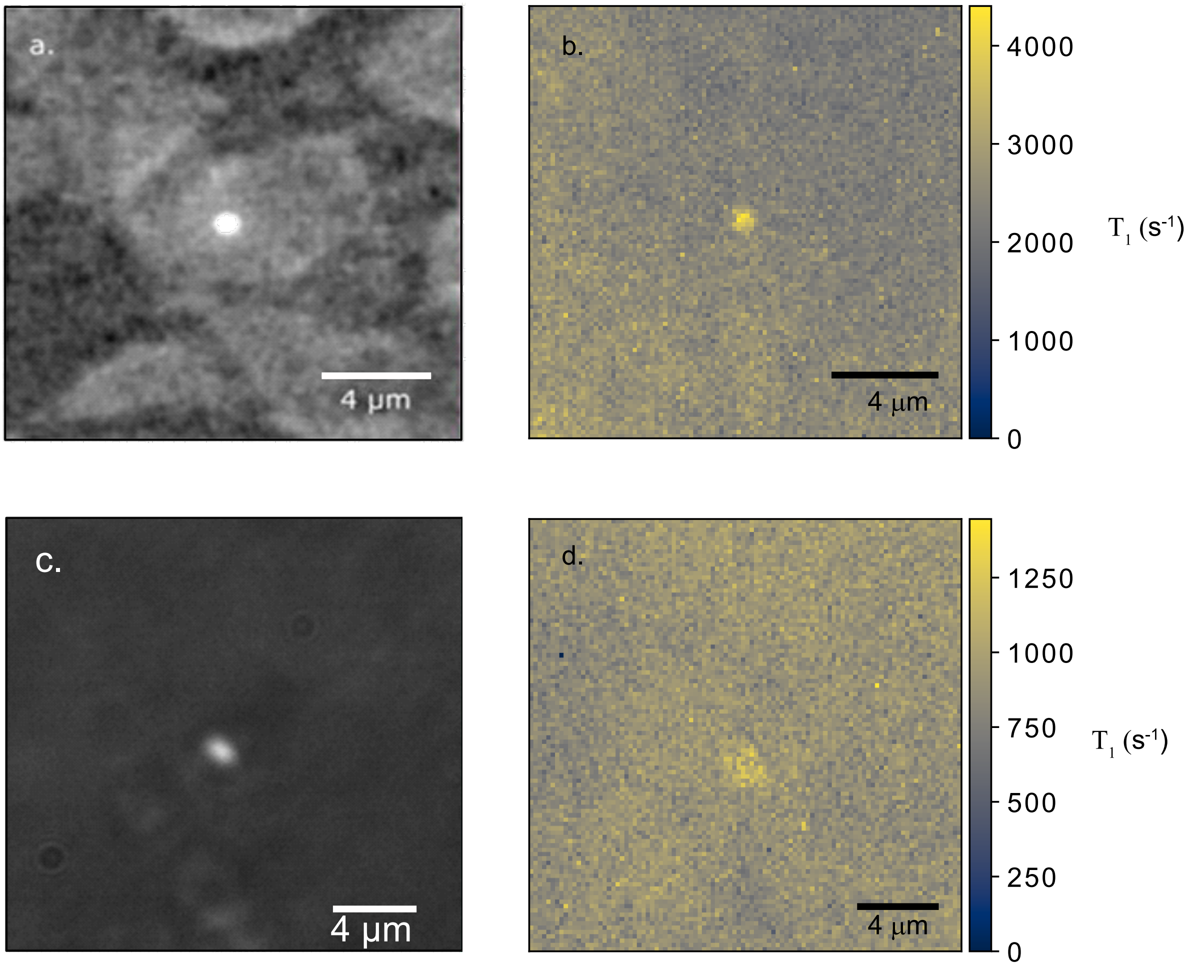

Fig. S4. T_1 images correlated with the positions of two cuticulosomes. (A) SEM image of a cuticulosome in a hair cell within the lagena. The cuticulosome presents as a bright region due to the electron-dense iron granules contained. (B) T_1 image taken from the same region as A. The NVs closest to the cuticulosome exhibit smaller T_1 times than the others due to the magnetic noise emanating from the cuticulosome. (C) Optical brightfield image of a cuticulosome taken from a hair cell within the basilar papilla of a pigeon. (D) T_1 image taken from the NV centers in the same field of view as C.

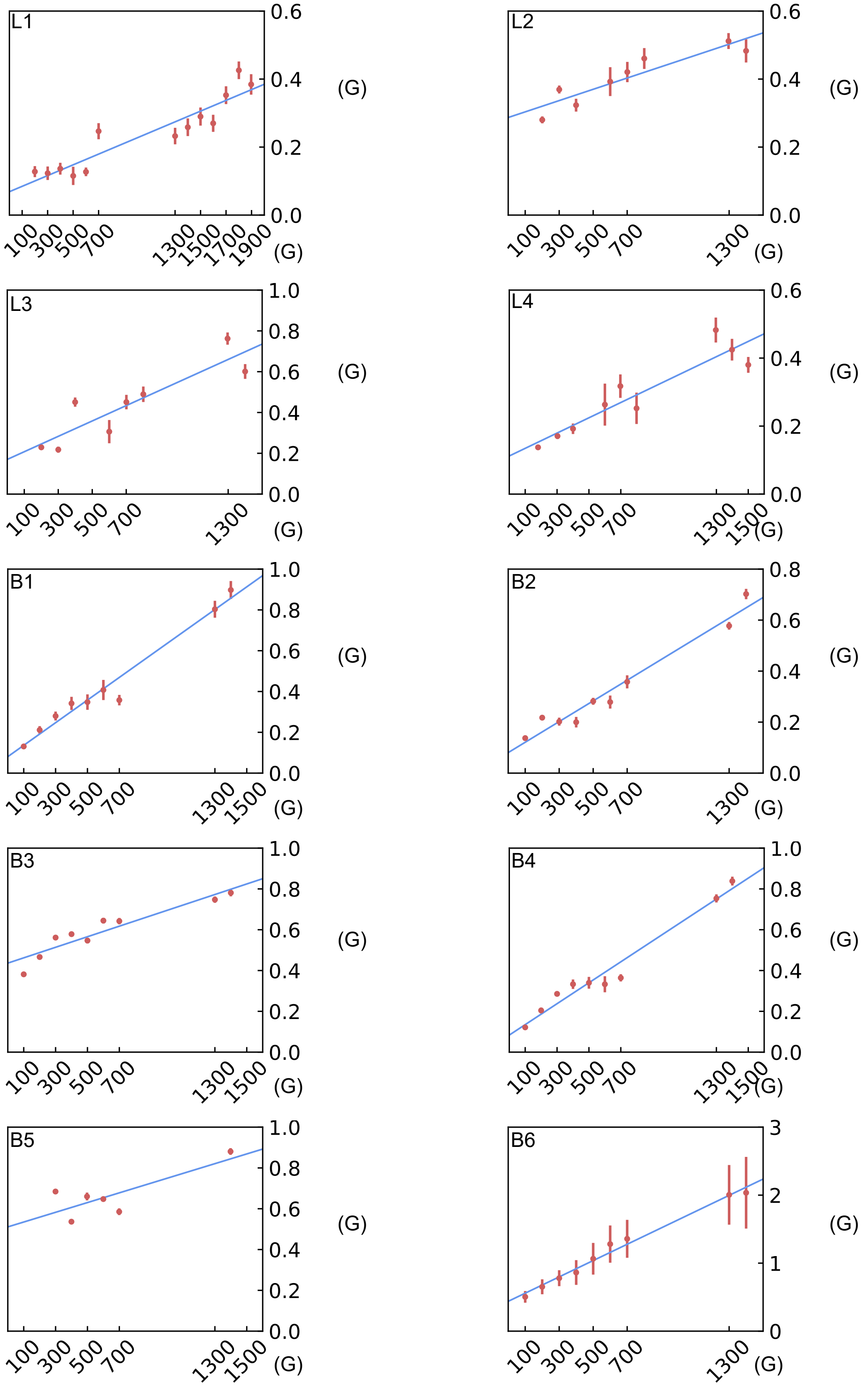

Fig. S5. Plots of the signal size against the applied field for each measured particle respectively. The linear fit to the data, ξ, is proportional to the magnetic susceptibility.

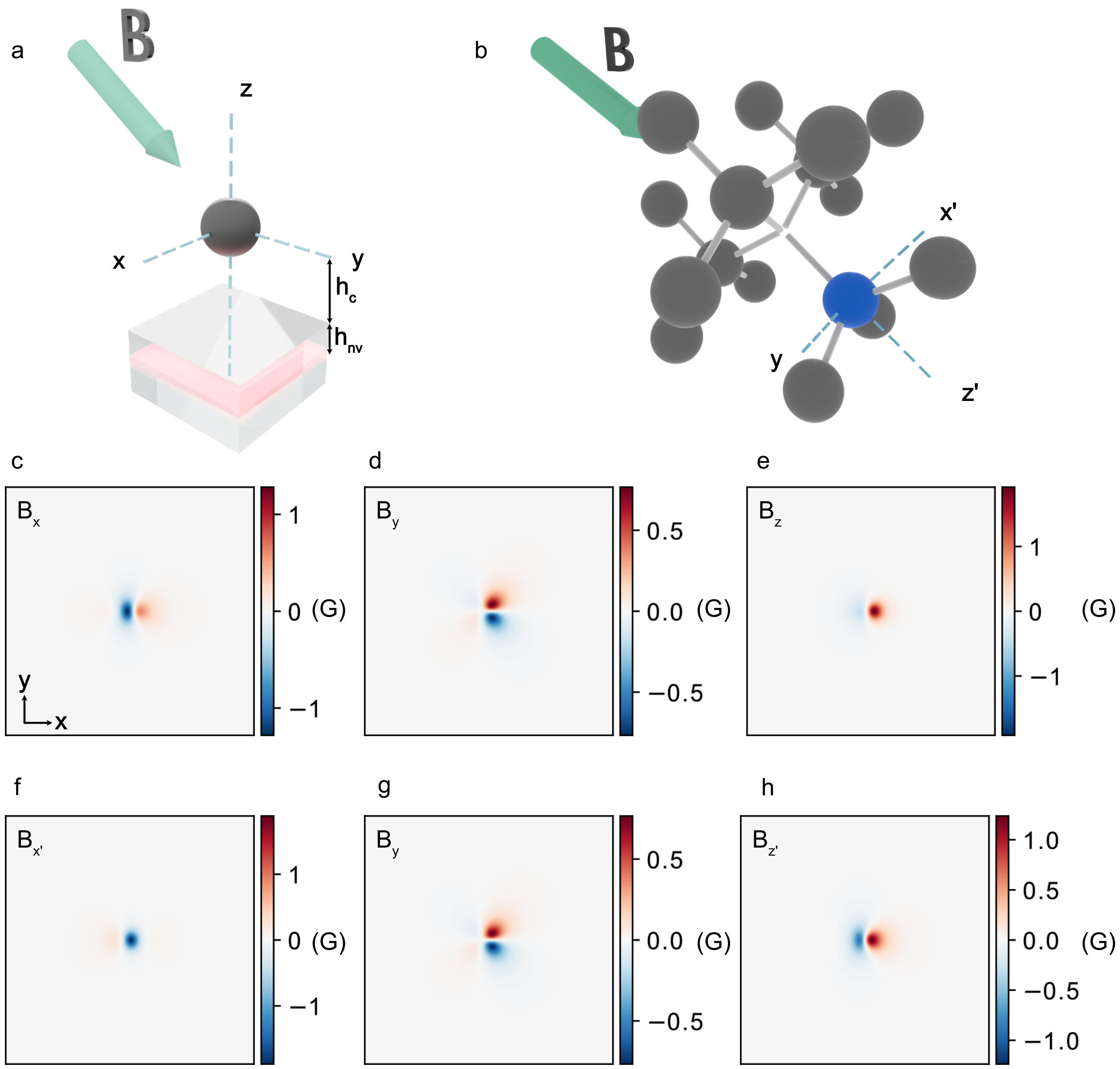

Fig. S6. Coordinate frames used in the theoretical model. (A) Schematic illustrating the applied magnetic field in the laboratory coordinate frame. The cuticulosome shown has a height of h_c from the diamond surface and the NV layer has a depth of h_{nv}. (B) Schematic of the applied magnetic field in the NV coordiate frame. The coordinate frame is rotated such that the z' axis is aligned along one of the four diamond crystallographic axes. (C-E) The x, y and z components of the stray magnetic field from a cuticulosome in the laboratory frame. (F-H) The x, y and z components of the same stray magnetic field in the reference frame of an NV center. The side lengths of all images are 4 µm

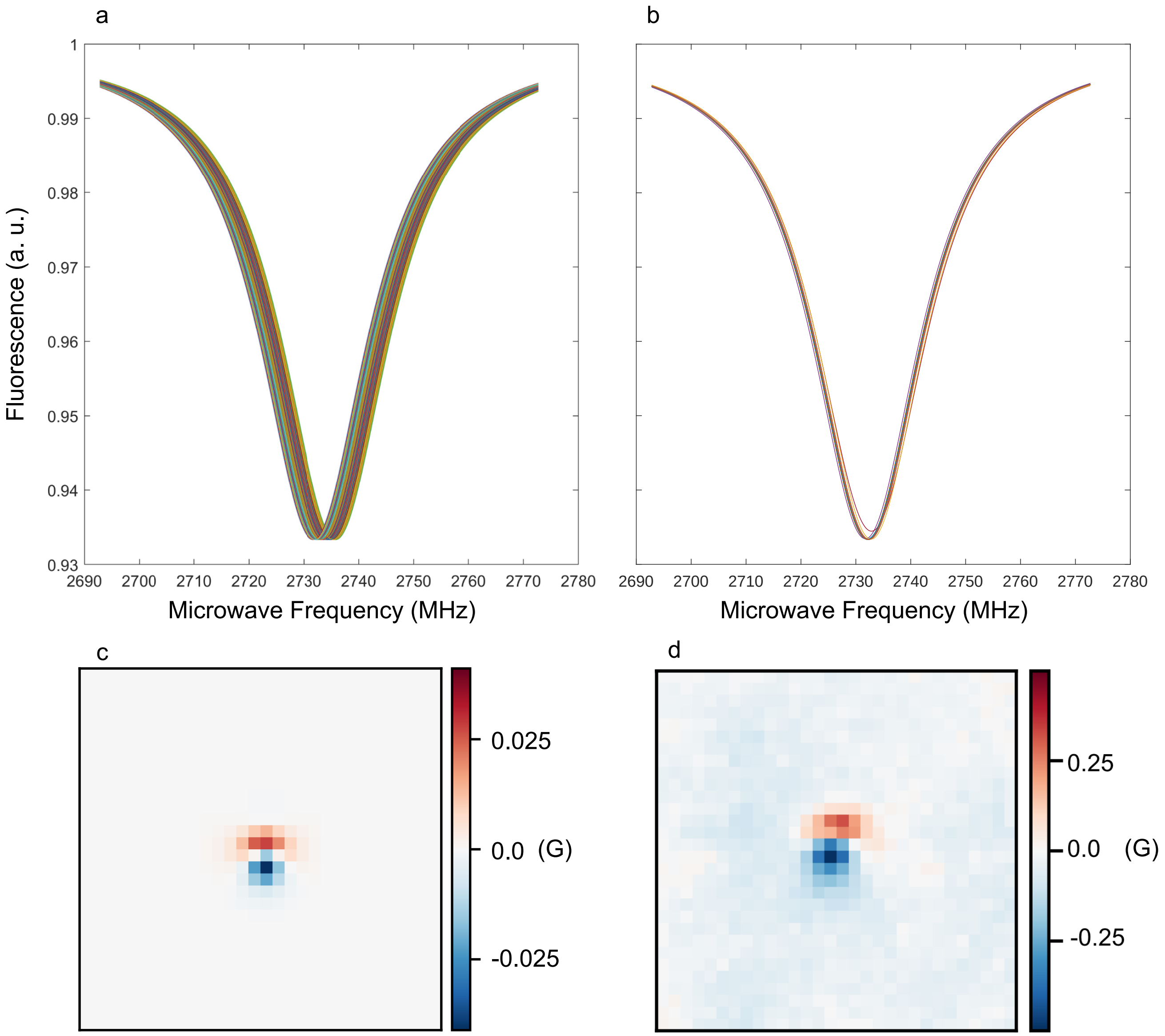

Fig. S7. Derivation of simulated images from the response of each NV in each pixel. (A) Each spectrum is the response from a simulated NV center in a single pixel beneath the cuticulosome. (B) Each Lorentzian represents the simulated quantum magnetic microscope response to the magnetic signal for a particular pixel. This is calculated by summing the simulated responses of each NV in that pixel. (C) Simulated magnetic field map using fitting parameters extracted from B. The side length of the plot is 13 µm. (D) An experimental comparison showing good qualitative agreement with the theoretical model. The side length of the plot is 13 µm.

Particle	ξ
L1	1.6×10^{-4}
L2	1.7×10^{-4}
L3	3.8×10^{-4}
L4	2.2×10^{-4}
B1	5.5×10^{-4}
B2	4.1×10^{-4}
B3	2.6×10^{-4}
B4	5.1×10^{-4}
B5	2.4×10^{-4}
B6	1.2×10^{-3}

Table S1. Summary of the ξ values for each particle measured in the pigeon sections.

References

1. A Dréau, et al., Avoiding power broadening in optically detected magnetic resonance of single NV defects for enhanced dc magnetic field sensitivity. *Phys. Rev. B* **84**, 195204 (2011).
2. JP Tetienne, et al., Proximity-Induced Artefacts in Magnetic Imaging with Nitrogen-Vacancy Ensembles in Diamond. *Sensors* **18**, 1290 (2018).
3. M Capelli, et al., Increased nitrogen-vacancy centre creation yield in diamond through electron beam irradiation at high temperature. *Carbon* **143**, 714–719 (2019).
